# Health Navigator Perspectives on Implementation of Healthy Michigan Plan Work Requirements

**DOI:** 10.1001/jamahealthforum.2022.1502

**Published:** 2022-06-10

**Authors:** R. Patrick Kelly, Gabriela Marcu, Amber Hardin, Samantha Iovan, Renuka Tipirneni

**Affiliations:** 1 Michigan Medicine, University of Michigan, Ann Arbor; 2 School of Information, University of Michigan, Ann Arbor; 3 Center for Health and Research Transformation, Ann Arbor, Michigan; 4 Institute for Healthcare Policy and Innovation, University of Michigan, Ann Arbor; 5 Division of General Medicine, Department of Internal Medicine, University of Michigan, Ann Arbor

## Abstract

**Importance:**

In 2020, Michigan Medicaid implemented a new work requirement policy for enrollees in the Healthy Michigan Plan (HMP), the state’s expanded Medicaid program. Under the policy, enrollees would be required to report 80 hours of work or other qualifying activities each month or obtain an exemption to maintain health insurance coverage.

**Objective:**

To assess the experiences of health navigators (and beneficiaries by proxy) with the implementation of Michigan's Medicaid work requirement.

**Design, Setting, and Participants:**

This qualitative study included health navigators who participated in 11 focus groups from September to December 2020. Focus group questions addressed topics related to implementation and communication of the HMP work requirement policy. A survey was administered to capture background information, assess readiness to assist with navigation, and assess the quality of the state’s communication about the policy.

**Main Outcomes and Measures:**

The main outcomes were general understanding of policy components, communications about the policy, navigation of the exemption process, navigation of the reporting process, and beneficiaries’ ability to comply with the policy.

**Results:**

Of 50 health navigators who participated in the focus groups, 44 (88.0%) responded to the survey and 43 provided demographic information (mean [SD] age, 44.0 [10.5] years). All 43 had at least some college or vocational education, with 27 (62.8%) reporting a 4-year degree or higher, and they resided in geographic regions across Michigan. Navigators indicated that they felt prepared to assist HMP enrollees navigate work requirements. Communication to beneficiaries regarding the new policy was viewed as an improvement compared with traditional Medicaid communications. However, the complex policy was potentially difficult for beneficiaries to understand. Limited language options besides English created barriers for some enrollees. Exemptions were confusing for beneficiaries owing to the numerous categories and sometimes vague guidelines. Those who used the online reporting system generally found the platform to be user-friendly. Navigators expressed concern regarding HMP enrollees’ ability to report compliance with work requirements owing to barriers with internet access, telephone access, language challenges, and computer literacy.

**Conclusions and Relevance:**

In this qualitative analysis of health navigators in Michigan, respondents reported overall positive feedback about many of the implementation and communication strategies, including human-centered design, automatic exemptions, and use of state administrative data to assess whether beneficiaries were in compliance with the policy; however, barriers to reporting remained for enrollees. The findings suggest that state agencies should seek to reduce administrative barriers and apply a human-centered design approach to alleviate risk of unnecessary loss of Medicaid benefits.

## Key Points

**Question:**

What were the experiences of health navigators and Michigan Medicaid expansion beneficiaries with the Healthy Michigan Plan work requirement policy in 2020?

**Findings:**

In this qualitative study of 50 health navigators in Michigan, respondents provided positive feedback about implementation of Medicaid work requirements, which used a human-centered design approach. Communication regarding the policy was viewed as improved compared with traditional Medicaid communications, and reporting systems appeared to be more user-friendly; the complexity of the policy and limited language options were barriers to compliance.

**Meaning:**

The findings suggest that state agencies should seek to reduce administrative barriers and apply a human-centered design approach to alleviate risk of unnecessary loss of Medicaid benefits.

## Teaser

This qualitative study describes perspectives of community health navigators about the work requirement policy of the Healthy Michigan Plan and experiences of Medicaid beneficiaries with the policy.

## Introduction

Michigan’s expanded Medicaid program, the Healthy Michigan Plan (HMP), has provided health insurance coverage to hundreds of thousands of Michigan residents since its launch in April 2014.^1,2^ As of January 2020, approximately 687 000 individuals were enrolled in HMP, and during the COVID-19 pandemic, this number increased to more than 900 000, representing approximately 10% of the state’s population.^3,4,5^ Michigan is 1 of 7 states in the US to pursue Medicaid expansion through a Section 1115 waiver, most often pursued to allow alternative features of state programs.^6^ Work requirements were one of the most recent features of several state Medicaid Section 1115 waivers to be approved by the Centers for Medicare & Medicaid Services. In 2018, the Michigan Legislature passed Public Act 208,^7^ adding workforce engagement requirements as a condition of eligibility for HMP enrollment. Michigan Medicaid implemented the new work requirement policy in January 2020.^7,8^ Under the new policy, many HMP enrollees were required to report 80 hours of work or other qualifying activities (eg, education, job training, or job searching) each month or obtain an exemption to maintain health insurance coverage.^9^ On March 4, 2020, a federal judge ruled that federal approval of the HMP work requirement was not consistent with the Medicaid statute because work requirements did not promote objectives of the Medicaid program, namely to provide coverage, and implementation of the policy was halted.^10^

Although many HMP enrollees would likely have been in compliance,^11,12^ implementing the work requirement policy could have created administrative barriers to maintaining enrollment.^13^ In the month before implementation, after an extensive process in which the state deemed many enrollees to be in compliance with the policy or automatically exempted many enrollees, the state reported that 238 000 HMP members would be subject to monthly work requirement reporting.^14^

Research on the Medicaid work requirement policy in Arkansas suggests that many enrollees were unaware of or confused by the policy before implementation was halted by the federal courts.^15^ Many enrollees experienced barriers to successful reporting, such as lack of internet access or difficulties setting up an account and reporting hours in the state’s system.^16,17^ Up to 18 000 individuals in Arkansas lost Medicaid coverage in 2018 owing to noncompliance.^18,19^ Medicaid enrollees in New Hampshire experienced similar barriers, and after the first month of implementation, only 32% of beneficiaries were in compliance with the state’s work requirement policy.^20^

Loss of Medicaid coverage may be associated with negative long-term consequences for the health and financial stability of enrollees and their families. Some individuals may switch to other types of coverage; however, many may remain uninsured for a period, and gaps in coverage are associated with delays in care and difficulties paying medical bills.^21^ In addition to the individual consequences of coverage losses, there are serious implications for health systems, safety net organizations, and communities. Before implementation, researchers estimated that Michigan hospitals could experience a 118% increase in uncompensated care costs and a 1.5% decrease in hospital operating margins as a result of coverage losses related to work requirements.^22^

To address anticipated barriers that HMP beneficiaries could experience after implementation of new work requirement rules, the Michigan Department of Health and Human Services (MDHHS) contracted with several organizations to evaluate and improve the state’s beneficiary communication and reporting systems. In the lead up to implementation, these organizations used a human-centered design approach, which involved direct engagement with the target audience (in this case, enrollees) to elicit their input on the design of communications and reporting systems to identify changes that would make these systems easier to use. During implementation, we partnered with the MDHHS to investigate the impact of the changes resulting from the human-centered design process. We gathered the perspectives of community health navigators (hereafter referred to as *navigators*) regarding the implementation of the HMP work requirement policy. The objectives were to (1) assess the effectiveness of the MDHHS’s communication strategies for the new policy and (2) inform decision-making regarding the use of human-centered design for future Medicaid initiatives.

## Methods

### Focus Groups

In this qualitative study, from September to December 2020, navigators were invited to participate in virtual focus groups aimed at understanding the rollout and implementation of HMP work requirements and to provide a sense of the beneficiary experience (a focus group guide is given in eMethods 1 in the Supplement). Continuing the human-centered design approach used before policy implementation, the study team developed a focus group guide to elicit participants’ perceptions, experiences, and challenges about the communications and reporting systems. With use of a purposive approach, recruitment targeted individuals in health care and community-based organizations who helped populations with low income maintain benefits and would be able to provide valuable information on the questions of interest. Approximately 5 navigators were recruited in each of Michigan’s 10 main geographic regions based on the state’s prosperity regions (an initiative designed to encourage regional collaboration and leveraging of resources).^23,24^ Navigators were identified through several sources, including the navigator roster for the MDHHS online application system for benefits (MI Bridges) and health care partners (recruitment information is given in eMethods 2 in the Supplement). Respondents were offered a $25 financial incentive for participation. This study was given a “not regulated” determination by the University of Michigan institutional review board. Verbal informed consent was obtained from all focus group participants, including permission to record and publish deidentified statements. The study followed the Consolidated Criteria for Reporting Qualitative Research (COREQ) reporting guideline.

Digital audio recordings were used to transcribe conversations from the focus groups, and data were deidentified. NVivo 12 (QSR International) was used to organize and manage focus group data.^25^ We used inductive qualitative thematic analysis to identify key themes in the data.^26^ Two independent coders (R.P.K., A.H.) reached consensus over themes and coding structure. The study team reached thematic saturation when geographic representation from the different regions of the state was obtained, all transcripts were coded, clear regularities emerged, and information became redundant. Of note, an additional focus group was held in Detroit owing to initial low turnout and limited insights.

### Survey

A brief survey was administered to participants after the focus groups were concluded to collect background information and gather responses to 2 additional questions. “Overall, how well did the state communicate with community navigators regarding Healthy Michigan Plan work requirements?” and (2) “Did you feel prepared to assist beneficiaries navigate work requirements at the time of implementation?” Responses were on a 5-point scale: 1 indicated not well at all or not at all prepared; 2, slightly well or slightly prepared; 3, moderately well or moderately prepared; 4, very well or very prepared; and 5, extremely well or extremely prepared (the survey is given in eMethods 3 in the Supplement).

Descriptive statistics were applied to the survey responses. These quantitative data were then paired with qualitative data from the focus groups to provide a comprehensive examination of the navigator perspective on the state’s communication and implementation of HMP work requirements. Quantitative data were analyzed using Microsoft Excel, version 2201.

## Results

### Sample Characteristics

A mean of 5 focus group participants (range, 3-7 participants) were recruited in each of Michigan’s 10 prosperity regions for a total of 50 participants. Of these participants, 44 (88.0%) responded to the survey and 43 provided demographic information (mean [SD] age, 44.0 [10.5] years). All 43 had at least some college or vocational education, with 27 (62.8%) reporting a 4-year degree or higher, and they resided in geographic regions across Michigan. Respondents self-identified through the survey instrument with investigator-defined options as American Indian/Alaska Native (2 [4.7%]), Asian/Asian American (1 [2.3%]), Black/African American (3 [7.0%]), Hispanic/Latino (3 [7.0%]), White (34 [76.7%]), and multiracial (1 [2.3%]) (eTables 1 and 2 in the Supplement give more sample details).

### Qualitative Themes

#### General Understanding of Policy Components

Overall, respondents had a good understanding of the work requirement policy and were able to recall key components of the policy (such as the eligibility criteria, the number of hours required in a given reporting period, exemptions, qualifying activities, reporting methods, failed months, and potential loss of coverage). Survey responses supported this high-level qualitative finding, with navigators indicating that they felt prepared to assist beneficiaries at the time of implementation and more than two-thirds indicating they were extremely or very prepared (Figure).

**Figure.**
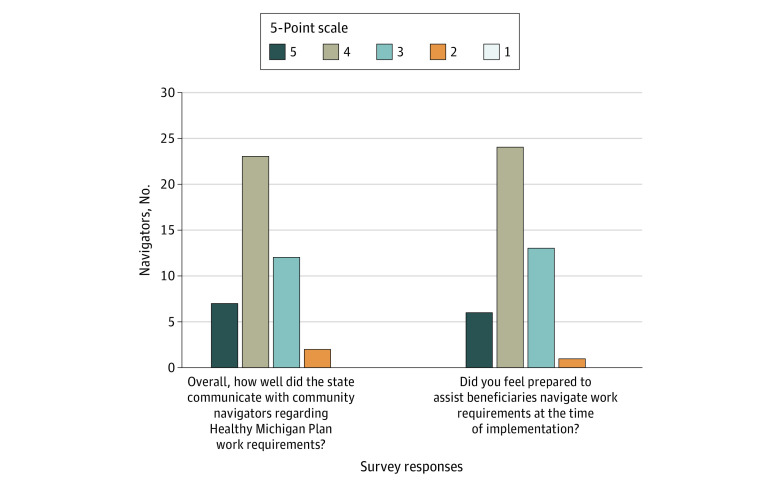
Navigators’ Perspectives on State Communication Related to Work Requirements and Their Own Preparedness to Assist Beneficiaries With Navigation Responses were on a 5-point scale: 1 indicated not well at all or not at all prepared; 2, slightly well or slightly prepared; 3, moderately well or moderately prepared; 4, very well or very prepared; and 5, extremely well or extremely prepared.

#### State Communication About Work Requirements and Educational Resources

Overall, navigators noted improvement in communications and educational opportunities about HMP work requirements compared with other Medicaid initiatives (which were marked by generic-looking envelopes and letters with large amounts of text and technical language, making it difficult for beneficiaries to realize the importance of the communication or interpret key information). Similarly, survey responses showed that more than two-thirds of the navigators felt that the state did extremely or very well in communicating information about work requirements. A total of 12 of 44 survey respondents (30%) indicated that the state communicated moderately well (Figure).

Box 1 gives a summary of key themes and representative quotes related to the state’s communication efforts. Many navigators perceived that HMP beneficiaries did not open their mail regularly and that traditional form letters from the state were often ignored by beneficiaries. Therefore, they believed the color-coded letters that resulted from the state’s human-centered design efforts helped draw attention and make the letters seem important. Many beneficiaries brought the letters with them when meeting with navigators. The navigators were also able to give beneficiaries advanced warning that the letters were coming, and the unique colors provided a useful point of reference. Navigators also indicated that the choice of color could serve as a visual cue to indicate the nature or urgency of the communication. The design of the letters, including language, formatting, and icons, were reported to be improved compared with traditional MDHHS communications to beneficiaries. Respondents positively referenced action-oriented headers, increased use of white space, and icons such as exclamation points. Navigators were mixed in their assessment of the appropriateness of the reading level of beneficiary communications. Respondents indicated that despite improvements to communication materials, the policy was inherently complex and difficult for HMP enrollees to understand.

Box 1. **Key Themes and Representative Quotes Regarding State Communication Around Medicaid Work Requirements**

**Key Themes**

- Perception that many beneficiaries may discard or ignore form letters sent from the MDHHS.
- The MDHHS’s work requirements–related communications were generally viewed as an improvement compared with traditional Medicaid communications.
- Improvements included
  - Color-coding and use of symbols on letters and envelopes to convey importance
  - Changes to formatting and white space to improve readability
  - Changes to language to be at a more appropriate literacy level
- Perception that the large amount of letters sent to beneficiaries over an extended period created confusion about what was important, leading some to ignore or discard letters.^a^
- Despite changes, the policy-related information contained in the letters could be difficult for many beneficiaries to process on their own and may have required assistance of navigators.

**Representative Quotes**

“I would say the same with the color-coordinating. I work primarily with clients with disabilities. But still, they all recognize and communicate differently. So, even colors, you know, red obviously stops. There’s something going on. Yellow, caution. So, I appreciated the inclusiveness of using different formats than just the black and white ‘Oh, here’s another form.’ And some clients are like, ‘Ugh, it’s another thing from MDHHS.’ Toss it aside. I was really happy that they changed the format so that the individual would understand, ‘Hey, this is important. Don’t throw it aside.’ And very different from other forms, for sure. So, I thought that was good.”
“I will say Medicaid did a good thing in having those bright yellow envelopes so that they actually paid attention to getting it as mail, which helped them come in and say, ‘I don’t understand what’s happening.’”
“MDHHS paperwork can be kind of confusing to just the average person, let alone for us who work with this all day long, but I did think it was laid out well, and that it was understandable.”
“They did try to make an effort at simplifying the language and making it really distinct.”
“Our community [has] a language barrier. So whatever they receive, they rush to ask us to read for them....They did not understand clearly what was going on because they could not read right or explain it right….They need accurate information that they can understand.”

Abbreviation: MDHHS, Michigan Department of Health and Human Services.

^a^
The MDHHS was partially constrained in its ability to control the number and timing of letters by federal waiver and regulatory requirements. The MDHHS was required to provide notice to beneficiaries about the new requirements several months in advance of implementation and to send a notice to beneficiaries if they did not currently qualify for an exemption.

Although there was great need to communicate the new work requirement policy to beneficiaries, many navigators stated that the volume of letters was overwhelming to clients. There was a perception that many letters were unnecessary, duplicative, or repetitive and that as a result, HMP enrollees had difficulty determining what was important, leading some to ignore or discard letters. Some respondents who served beneficiaries who did not speak English also indicated that communications to beneficiaries in their communities were lacking because only mailings in English were sent. Of note, the MDHHS included information on how beneficiaries could access free language assistance services in a variety of languages.

#### Navigation of the Exemption Process

In September 2019, the MDHHS issued notices to beneficiaries to indicate whether they were subject to the new policy or had an automatic exemption. Exemptions were given for numerous reasons including medical conditions, disabilities, and pregnancies.^27^ For beneficiaries required to report an exemption manually, the MDHHS offered 3 methods to report, including online (MI Bridges), by telephone, or in person. Navigators expressed confusion regarding the exemption process, reporting that the exemption categories were numerous and seemed vague (such as the “medically frail” category). Several navigators noted that beneficiaries often did not recognize they had health conditions that might make them exempt from the work requirement policy. Box 2 details key themes and representative quotes related to the exemption process.

Box 2. **Key Themes and Representative Quotes Regarding Navigation of the Work Requirement Exemption Process**

**Key Themes**

- Exemptions were confusing for beneficiaries owing to the numerous categories to consider and some reports of vague guidelines associated with the categories, such as the medically frail exemption.^a^
- Navigators reported limited feedback or notification that an exemption had been received or processed.
- Beneficiaries experienced confusion related to letters received and whether or not they were automatically exempt, should report an exemption, or would need to report work-related activities monthly.

**Representative Quotes**

“I think there was a lot of confusion from clients about what constituted an exemption. So, a lot of it was going over, reading it through and really asking them questions to see if they qualified for one. A lot of people were worried that they needed…a doctor’s note…and I said, ‘That can help, but right now, they’re not asking for that. You can get that in the future if they need it, but you don’t need it up front.’ A lot of people were worried they needed something right away, but I helped explain to them that they didn’t at the moment….But a lot of people had a lot of ongoing health conditions that prevented them from working, but they weren’t necessarily getting disability. So, helping people determine whether or not they were exempt was a little bit complicated because it was subjective but also true to their life. There was a reason they weren’t working. It was just hard for them, I guess, to grapple with it when it was presented in front of them in that way.”
“They recognized there was a confusion between exemptions for things like disabilities vs sort of alternatives to the work requirements, or kind of the terminology. But it’s hard for me to say how that was received by the clients because again, I didn’t get a lot of feedback. I think just because there was…so many different things that you could respond with, so many different exemptions, it was complicated. And the people who were implementing this, obviously…they weren’t responsible for that. That was legislation and policy writing that came up with that, so they had to try to make that as workable as possible. So, I think it was kind of complicated to begin with, and that was the real hard part. But in terms of trying to communicate it, I feel like they probably did as best they could do.”

^a^
The Michigan Department of Health and Human Services began issuing notices to beneficiaries to indicate whether they had to follow the new requirements (letter included exemption form) or they had an automatic exemption in fall 2019. Exemptions were given for numerous reasons, including medical conditions, disabilities, and pregnancies.

#### Navigation of the Work Reporting Process

Box 3 shows key themes and representative quotes regarding the work reporting process. Most navigators were unable to provide substantive feedback on the 3 MDHHS systems (MI Bridges, telephone, in person) used to report work hours owing to the limited time frame of reporting before the halt of the policy’s implementation. For navigators who had interacted with the reporting systems, MI Bridges was often their preferred method. Navigation of MI Bridges was generally considered to be user-friendly. The online platform provided users the opportunity to print confirmation of work requirement reporting (proof that provided reassurance that reporting was successful). Several navigators spoke about the challenges that beneficiaries who did not speak English experienced when using MI Bridges because the platform does not have multiple language options. There were additional problems for non-English–speaking beneficiaries when using the telephone reporting system and the help line related to the available dialects.

Box 3. **Key Themes and Representative Quotes Regarding Navigation of the Work Reporting Process**

**Key Themes**

- Respondents provided limited feedback on work reporting systems given the short period of implementation.
- Those with experience interacting with the MI Bridges online reporting system generally found the platform to be user friendly.^a^
- Navigators expressed concern regarding enrollees’ ability to report owing to barriers with internet access, telephone access (including limited minutes), language challenges, and computer literacy.

**Representative Quotes**

“I never actually assisted anyone with the actual reporting, so it’s hard. I don’t know if that’s a good thing or a bad thing. I don’t know if that means that people were doing it on their own and felt comfortable, or if people at that point had just given up and were like, ‘this isn’t going to work for me.’”
“We felt that everything that had been implemented in that regard was very easy. It was user-friendly. It was extremely simple as far as going into reporting, and you’d have the navigation pane on the left side of the page where you would just, you know, ‘Do you have an exemption?’ or ‘Do you want to report?’ And you just kind of clicked a couple buttons. It wasn’t very detailed. You didn’t have to provide a lot of additional information. It was just, if you’re reporting that you were working, ‘I’m employed at 40 hours,’ or ‘I have an exemption, and this is what it is.’ There was, I think, a small comments box where you could add a little bit of additional information. But no, it was pretty user-friendly. One of the better things that’s user-friendly on MI Bridges.”
“The work requirement issue would put a lot extra work on our benefits coordinator. I mean, we basically hand-hold a lot of the applications the way it is and trying to keep them on Medicaid. But then, this extra added on…would be a lot of work for her to be able to keep up with all the work requirements reporting on all these people because they don’t have the transportation. A lot of them don’t have phones. They have those–they call them ‘Obama phones.’ And they run out of minutes. And it would add an extra burden onto her position to try to keep things straight and to keep the reporting going.”
“Doing automated phone calls is often really hard, even if people have enough English to speak, it’s very different listening on the phone and trying to understand automated prompts. You don’t see the person’s mouth move. It’s very fast. Even when Russian translators are available—for instance, when they call their Healthy Michigan program, it’s very hard to get to the point where you can ask for a Russian translator. So, I mostly helped them understand how to do it over MI Bridges or just encouraged them to come in, and I would help them each month if they needed the help each month because it was a difficult process for them.”
“The thing is on top of the language barrier, I think it’s the technical understanding or deficiency. Not everyone has the ability to use the computer or function with it. And not everyone—most of them—they do not have their own computer or laptops to function with it. So, it was the most easier way to make the trip and come to us to help them with it.”

^a^
MI Bridges is the Michigan Department of Health and Human Services online application system for benefits.

#### Perspectives of Beneficiaries Regarding Their Ability to Comply With the Policy

As shown in Box 4, most navigators recounted concerns expressed by beneficiaries, including fear about loss of coverage and corresponding negative health consequences, challenges with finding work, and barriers to getting sufficient hours. Navigators noted that the expectation of monthly reporting to remain in compliance presented a substantial challenge for HMP enrollees. This issue was compounded when clients faced technological barriers, including difficulties with internet access, access to telephones or cellular minutes, and low computer literacy.

Box 4. **Key Themes and Representative Quotes on Perspectives of Beneficiaries Regarding Their Ability to Comply With the Policy**

**Key Themes**

- Although the work requirement halted before beneficiaries could be disenrolled for failure to report work hours, there was a strong belief among navigators that many of their clients would be disenrolled and would experience negative health and financial consequences from being uninsured.
- Perceived challenges to compliance included
  - Ability to find work (and ability to obtain work if a client was not employed recently) and barriers to being able to obtain sufficient hours.
  - The expectation of monthly reporting.
  - Technological barriers such as internet access, access to telephones (and limited minute cellular phones), and low computer literacy.
  - Confusion among beneficiaries about whether they were receiving HMP because many only recognized their coverage through the name of their managed care plan.
  - Barriers associated with social determinants of health, including transportation options; access to educational, economic, and job opportunities; access to health care services; and social supports.
  - Cultural and language barriers in communities with large immigrant populations in which English is not the first language.
- Navigators anticipated an increase in the uninsured rate in Michigan and an increasing number of people seeking uncompensated care, which could have negative economic consequences.
- A few navigators indicated that they worked with beneficiaries who were not particularly concerned about their ability to comply with the work requirements (eg, students, younger adults, and people who were exempt), and a few navigators implied that they were comfortable with work being tied to health insurance coverage.
- Many navigators indicated that they were relieved when the work requirements were halted because of the negative effects they would likely have had in their patient population.

**Representative Quotes**

“In the Upper Peninsula here, there are very few jobs that people who haven’t worked in years would be able to get….I spoke with one employer, and he said that they have, like, 80 employees at the local McDonald’s. And he said that they would have a problem just trying to find enough work for them when they have 80 employees already.”
“It’s a lot of reporting when it’s hard to keep people on [HMP], because they have to renew every year.”
“One of the problems that our clients faced was that the dialect that the person was speaking on the phone is completely different from the dialect that they understand. So, that wasn’t helpful at all sometimes….I tried calling myself three times, and every single time I got somebody who spoke the Egyptian dialect….In our community, it’s mostly Lebanese, Iraqi, and Yemeni individuals. So, those are 3 different dialects, and none of those were ever on the call when I called.”
“A lot of our patient population don’t think of it in terms of Healthy Michigan Plan. They see that, and they don’t really know that that refers to the coverage that they have. They talk about things like, ‘I have Molina, I have McLaren, I have Priority Health.’ Even though MDHHS did a great job with the design of the letters that went out, there still, for a lot of people, wasn’t something that got their attention that ‘This actually applies to something I have right now,’ because they don’t conceptualize their coverage as Healthy Michigan, that umbrella; they think about it in terms of the particular plan that they’re under.”
“For us as a health care facility…it would have had a financial impact on us for patients…who don’t want to comply. You know, they’ll get cut off, which is fine, but they still want to seek care. And we do have programs that we could help them with their bill. But, you know, one of the things with seeing Medicaid patients as a Federally Qualified Health Center is we’re actually able to meet our cost if we see a Medicaid patient. If we see a patient who is uninsured, we don’t get our cost…it is a financial impact on us…it does have an adverse effect on the patients who choose not to [comply] and they feel like they either will just get off Medicaid and then seek care and not pay their bill, or not seek care at all and then have long-term consequences for not seeking care.”

Abbreviations: HMP, Healthy Michigan Plan; MDHHS, Michigan Department of Health and Human Services.

Navigators also expressed concern that many beneficiaries may not have been aware of the work requirement changes despite the MDHHS communications. A persistent concern was that many HMP beneficiaries did not recognize that they were enrolled in HMP, which likely created barriers to compliance with reporting. Other potential barriers to compliance with the work requirement policy identified by navigators included limited transportation; lack of access to educational, economic, and job opportunities; lack of access to health care services and social supports; and language barriers.

## Discussion

In this qualitative study conducted after implementation of a state Medicaid work requirement, navigators indicated that they overall felt prepared to assist HMP beneficiaries with the newly imposed work requirement. The state’s educational materials and community learning opportunities received positive feedback and were viewed by navigators as an improvement compared with traditional communications. Owing to the short period of implementation, respondents did not have many experiences with helping beneficiaries report work hours. However, the limited feedback provided in the focus groups suggests that the systems set up by the state seemed user-friendly. These findings suggest that the MDHHS’s efforts to apply human-centered design to communications and reporting systems were beneficial.

Regardless, a policy such as a work requirement policy is inherently complicated. Even though communications were viewed positively, the new work requirement policy was difficult to navigate for trained navigators and for HMP enrollees, who experienced challenges including difficulties with internet and telephone access, transportation barriers, limited employment opportunities, and other social factors. Although HMP work requirements were halted before beneficiaries could be disenrolled for failure to report work hours, navigators expressed a strong belief that many would be disenrolled and subsequently experience negative health and financial consequences. In Arkansas, disenrollment occurred before the policy was overturned by the federal courts. In literature before Medicaid expansion under the Patient Protection and Affordable Care Act, uninsurance, including gain, loss, or change in coverage, was shown to be associated with negative health outcomes, including for individuals with chronic health conditions.^28,29^

There was substantial use of resources and concerted efforts by the MDHHS to communicate clearly information about the work requirement policy and to make the policy easy to navigate. Despite this, reports indicate that approximately 80 000 beneficiaries could have lost their Medicaid coverage if they had failed to report work hours in February and March 2020.^30^

These early findings from states implementing Medicaid work requirements appear to be consistent with the experience of work requirements previously instituted under the Supplemental Nutrition Assistance Program and the Temporary Assistance for Needy Families program. In prior assessments of the work requirements of those programs, there was no evidence of sustained improvements in employment or socioeconomic status among beneficiaries, calling into question the utility of such policies.^31,32,33,34,35^ Similarly, Sommers and Allen^36^ found that implementation of Medicaid work requirements in Arkansas was not associated with substantial increases in employment, hours worked, and community engagement activities. After 1 year of implementation of the work requirement policy, there were more uninsured Arkansas residents and no changes in relevant economic outcomes.^36,37,38^

### Policy Implications

The study findings may have important implications for various stakeholders in states implementing work requirements and other new Medicaid policies. For beneficiaries, losing Medicaid coverage may be associated with negative long-term consequences for the health and financial stability of enrollees and their families.^39,40,41^ Prior evidence suggests that people are healthier and more productive when they have health insurance coverage.^29^ In a previous survey, beneficiaries who reported that their health improved after gaining HMP coverage were 4 times as likely to say that they were doing a better job at work.^11^

In addition to the individual consequences of coverage loss, there may be important implications for health systems, safety net organizations, and communities. A study by Haught et al^22^ found that hospitals may experience weakened financial status owing to increases in uncompensated care. Moreover, the authors described the potential for lower revenue leading to hospital closures, particularly in rural areas, which could have adverse economic effects in communities.^22,41^

For Medicaid and other government agencies, several lessons may be applied when implementing new policies in the future. To apply the policy passed by the Michigan state legislature fairly to beneficiaries and reduce administrative burden, the state government tried to automate and streamline much of the work requirement process. The MDHHS deemed in compliance or automatically exempted hundreds of thousands of HMP enrollees using administrative data. We believe that Michigan and other states should consider expanding this practice to other state-level programs. More generally, states should look to shift the burden of reporting from the individual to the state government to reduce potential loss of benefits owing to barriers in reporting.^14^ In addition, the positive feedback from navigators suggests that a human-centered design approach to government communications and systems should be used by government administrators. An area for improvement for Michigan includes increasing the number of languages available for communication materials, reporting platforms, and telephone help lines.

### Limitations

This study has several limitations. Virtual focus groups were held approximately 6 to 9 months after the implementation of work requirements were halted, introducing the possibility of recall bias among respondents; however, the general understanding and recollection of the policy were high based on the responses gathered.

Data collection captured the perspectives of navigators as a proxy for the beneficiary experience. We chose to target navigators owing to concerns about our ability to reach HMP enrollees and engage them in a virtual focus group because of COVID-19–associated research restrictions. We also expected that navigators would be more likely to recall aspects of the HMP work requirements, given the short period of implementation, and therefore provide more robust feedback on the state’s related communications and systems.

## Conclusions

In this qualitative study of health navigators in Michigan, respondents reported overall positive feedback about many of the implementation and communication strategies used by the MDHHS. The State of Michigan took a number of steps to reduce potential administrative and communication barriers, including using a human-centered design, automatic exemptions, and deeming beneficiaries in compliance based on state administrative data. Although these steps were helpful for many HMP enrollees, evidence suggests barriers to compliance remained for many beneficiaries because approximately 80 000 HMP enrollees faced the prospect of coverage loss after March 2020. Medicaid work requirements were found to negatively affect health insurance coverage for populations with low income in other states.^17,18,19,20,21^ The findings suggest that state agencies should seek to eliminate or reduce administrative barriers and shift the burden of reporting from the individual to alternate modalities to alleviate risk of unnecessary loss of benefits. Application of a human-centered design approach may help reduce barriers to understanding new policies and compliance.

## Notes

Supplement. **eMethods 1.** Focus Group Guide **eMethods 2.** Recruitment Methods **eMethods 3.** Post-Focus Group Survey **eTable 1.** Focus Group Participants by Prosperity Region and Focus Group Time **eTable 2.** Sample Characteristics of Participants Who Completed Post Survey
