# Supplementary material for: Health Navigator Perspectives on Implementation of Healthy Michigan Plan Work Requirements
Source: JAMA Health Forum. 2022 Jun 10;3(6):e221502. doi: 10.1001/jamahealthforum.2022.1502 (PMC9187957; doi:10.1001/jamahealthforum.2022.1502)
Supplement: Supplement. — eMethods 1. Focus Group Guide eMethods 2. Recruitment Methods eMethods 3. Post-Focus Group Survey eTable 1. Focus Group Participants by Prosperity Region and Focus Group Time eTable 2. Sample Characteristics of Participants Who Completed Post Survey [file jamahealthforum-e221502-s001.pdf]

Supplemental Online Content

Kelly RP, Marcu G, Hardin A, Iovan S, Tipirneni R. Health navigator perspectives on implementation of Healthy Michigan Plan work requirements. *JAMA Health Forum.* 2022;3(6):e221502. doi:10.1001/jamahealthforum.2022.1502

eMethods 1. Focus Group Guide

eMethods 2. Recruitment Methods

eMethods 3. Post-Focus Group Survey

eTable 1. Focus Group Participants by Prosperity Region and Focus Group Time

eTable 2. Sample Characteristics of Participants Who Completed Post Survey

This supplemental material has been provided by the authors to give readers additional information about their work.

© 2022 Kelly RP et al. *JAMA Health Forum.*

eMethods 1: HMP Work Requirements Focus Group Guide

BACKGROUND
The State of Michigan adopted new work requirements for Healthy Michigan Plan enrollees, which took effect on January 1, 2020. On March 4, 2020, a federal judge ruled that approval of the HMP work requirements was unlawful. This ruling stopped MDHHS' implementation and enforcement of the work rules.

Guidelines:

- Speak one at a time
- Take space/make space
- Mute your device when not speaking
- We are recording the discussion
- There are no right or wrong answers

I. KNOWLEDGE OF WORK REQUIREMENTS POLICY

For this first section, we want to jog your minds about work requirements broadly and some of the more specific components you may have become familiar with.

1) **Can you tell us what you remember about the work requirements policy?**
   a) OPTIONAL PROBE: How often were beneficiaries supposed to report?
   b) OPTIONAL PROBE: What methods were available for reporting (online vs phone vs in-person)?
   c) OPTIONAL PROBE: What would happen if beneficiaries didn't report?
   d) OPTIONAL PROBE: Do you remember if beneficiaries were supposed to provide some type of proof or documentation when reporting?
   e) OPTIONAL PROBE: What might cause someone to lose coverage?
   f) OPTIONAL PROBE: Time frame for losing coverage?

II. MDHHS MESSAGING AND COMMUNICATION

In this next section, we'll focus on how you learned about work requirements

2) **How did you become aware of the new program requirements? When did you become aware of the changes? Did you go anywhere else to find more information?**
   a. OPTIONAL PROBE: educational materials, regional forums. MDHHS secondary items: webinars, radio ads, social media
   b. OPTIONAL PROBE: Messages from MDHHS, MHPs, other entities?

3) **Were there specific documents sent from the state that you found to be particularly helpful regarding your understanding of work requirements?**
   a. OPTIONAL PROBE: Were these materials useful to beneficiaries? Beneficiaries understanding?
   b. OPTIONAL PROBE: Were there major gaps in educational materials?

4) **Did you engage in any of the regional community forums or town halls organized by MDHHS in the fall of 2019? What was your experience? Did you provide feedback?**
   a. OPTIONAL PROBE: Other formats (e.g. affinity groups, webinars, etc.)?
   b. OPTIONAL PROBE: Would you participate in a future community forum based on your experience during the HMP forums?
   c. OPTIONAL PROBE: Did MDHHS present key information on the work requirements in a way that was accessible?
   d. OPTIONAL PROBE: Did MDHHS do a good job in publicizing the forums?

© 2022 Kelly RP et al. *JAMA Health Forum.*

5) **How did the state's communication regarding work requirements compare to other Medicaid communication from MDHHS?**

   a. OPTIONAL PROBE: How could the state's communications been improved?

<u>III. STAKEHOLDER EXPERIENCE ASSISTING WITH REPORTING</u>

Thank you for all of your responses in that last section. Next, we'd like to learn about experiences you may have had working with beneficiaries to navigate work requirements.

6) **Did any of you assist beneficiaries with reporting hours to comply with the Healthy Michigan Plan (or "Medicaid") work requirements? Can you tell us more about those experiences?**
   a. OPTIONAL PROBE: How did you try to report through…
      MI Bridges vs. phone line vs. in a local MDHHS office vs. other location
   b. OPTIONAL PROBE: What worked well with reporting? What was challenging? What would have made it less challenging? (e.g., length of time on hold?)
      MI Bridges vs. phone line vs. in a local MDHHS office

<u>IV. PERCEPTIONS OF BENEFICIARY EXPERIENCE WITH WORK REQUIREMENTS</u>

Next we'd like to get your perspective on the experience of beneficiaries attempting to comply with work requirements.

7) **What did you hear from beneficiaries about their ability to comply with work requirements? Do you have any specific stories to share?**

OPTIONAL PROBE: Work aspects vs reporting aspects? Concerns about loss of coverage?
OPTIONAL PROBE: Can you tell us more stories about beneficiaries attempting to navigate exemptions? E.g. What were the perspectives of beneficiaries in terms of whether they should qualify as medically frail?

<u>V. WRAP UP</u>

Thank you. The information you provided us will be very helpful for our work and the feedback we share with MDHHS.

8) **Is there anything else about your experience during the implementation of work requirements that you want to share?**

© 2022 Kelly RP et al. *JAMA Health Forum.*

eMethods 2: Recruitment Methods

Recruitment occurred through existing health care partnerships with the Center for Health Research and Transformation, Michigan Department of Health and Human Services, and Metro Health Foundation. The study team reviewed the state's navigator roster for the online application system for benefits (MI Bridges). These navigation partners aid MI Bridges users by managing cases, helping to connect beneficiaries to needed resources, and assisting with benefits applications. In addition, we identified community health workers in each region who may have assisted with navigation related to Medicaid work requirements through the Michigan Community Health Worker Alliance (MiCHWA). The Metro Health Foundation provided information for navigators in the Detroit Metro area, where they primarily operate, based on their network of grantees and partners. The study team used a purposive approach to sampling participants, seeking those who would be able to provide valuable information on the topic/question of interest and have a diverse set of experiences and perspectives. In recruiting participants from a wide range of agencies/organizations we aimed to capture a range of experiences, from more limited to more extensive understanding of the policy and with diverse types of beneficiaries assisted. Therefore, recruitment targeted individuals in health care and community-based organizations who help low-income populations maintain benefits. We reached out via email to contacts in each of these regions inviting them to participate.

© 2022 Kelly RP et al. *JAMA Health Forum.*

eMethods 3: HMP Work Requirements Post-Focus Group Survey

Thank you for your participation in the Healthy Michigan Plan work requirements focus group. We would like to collect some additional information from you. Your responses are confidential and your name and identifying information will not be linked to your answers given during the focus group.

Please make sure to complete the entire form (including your mailing address) so we can mail you your gift card incentive.

Q1 First, we'd like you to answer a couple of questions related to the rollout and communication around Healthy Michigan Plan work requirements.

Q2 Did you feel prepared to assist beneficiaries navigate work requirements at the time of implementation?

- ○ Extremely prepared (5)
- ○ Very prepared (4)
- ○ Moderately prepared (3)
- ○ Slightly prepared (2)
- ○ Not at all prepared (1)

Q3 Overall, how well did the state communicate with community navigators regarding Healthy Michigan Plan work requirements?

- ○ Extremely well (5)
- ○ Very well (4)
- ○ Moderately well (3)
- ○ Slightly well (2)
- ○ Not well at all (1)

Please help us better understand your background by responding to the next questions below.

Q4 What best describes your level of education? Please choose the one answer that best fits.

- ○ Less than high school
- ○ High school graduate
- ○ GED Program
- ○ Some college or technical/vocational training
- ○ 2 year degree
- ○ 4 year degree
- ○ Graduate/professional degree
- ○ Doctorate

Q5 What is your age?

____

Q6 Please select one or more of the following which best describes your race and ethnicity

- ☐ American Indian/Alaskan Native
- ☐ Asian/Asian American
- ☐ Black/African American
- ☐ Hawaiian Native/Pacific Islander
- ☐ Hispanic/Latino
- ☐ White
- ☐ Other (please describe) ____
- ☐ Unknown

© 2022 Kelly RP et al. *JAMA Health Forum.*

Q7 What organization do you work for?

____

Q8 Please enter your job title/role below

____

Lastly, in order to receive your gift card for participation in the work requirements focus group, we need to collect your name and mailing address. Please enter your information below. The gift card will be mailed to you in the coming weeks.

Q9 Name

____

Q10 Street Address

____

Q11 City

____

Q12 State

____

Q13 Postal Code

____

© 2022 Kelly RP et al. *JAMA Health Forum.*

Sample Characteristics

eTable 1. Focus group participants by prosperity region and focus group time

Prosperity Region	Number of Navigators Who Participated (N=50)	Focus Group Time*	Focus Group Response Rate**
**1. Upper Peninsula**	7 (14.0%)	48:20:00	100%
**2. Northwest**	7 (14.0%)	47:23:00	70%
**3. Northeast**	4 (8.0%)	42:43:00	44%
**4. West**	4 (8.0%)	55:54:00	33%
**5. East Central**	4 (8.0%)	53:37:00	44%
**6. East**	5 (10.0%)	50:28:00	28%
**7. South Central**	6 (12.0%)	53:17:00	67%
**8. Southwest**	4 (8.0%)	52:13:00	44%
**9. Southeast**	3 (6.0%)	35:15:00	38%
**10. Metro Detroit 1*** **	2 (4.0%)	50:52:00	8%
**10. Metro Detroit 2*** **	4 (8.0%)	1:25:23	44%

*Note: Mean focus group time was 52:19 with a SD of 11:50
**Note: Focus group response rate calculated by the # of focus group respondents/# of people invited to attend
***Note: Two focus groups were held with Metro Detroit due to low turnout in the first focus group for the region.

eTable 2. Sample characteristics of participants who completed post survey

Demographic Information	Navigators (N=44)
**Age, Mean (SD)**	44 (10.6)
**Race/Ethnicity (%)**	
American Indian/Alaskan Native	4.7%
Asian/Asian American	2.3%
Black/African American	7.0%
Hispanic/Latino	7.0%
White	76.7%
Multiracial	2.3%
**Education level (%)**	
Some college or technical/vocational training	20.9%
2-year degree	16.2%
4-year degree	32.6%
Graduate/professional degree	27.9%
Doctorate	2.3%

© 2022 Kelly RP et al. *JAMA Health Forum.*
